# Effects of the Preparation Conditions and Reinforcement Mechanism of Polyvinyl Acetate Soil Stabilizer

**DOI:** 10.3390/polym11030506

**Published:** 2019-03-17

**Authors:** Fan Bu, Jin Liu, Yuxia Bai, Debi Prasanna Kanungo, Zezhuo Song, Fanxuan Kong, Cheng Pan

**Affiliations:** 1 School of Earth Sciences and Engineering, Hohai University, Nanjing 210098, China; bf_hhu@163.com (F.B.); byxhhu@163.com (Y.B.); szzhhu@163.com (Z.S.); kfxhhu@163.com (F.K.); panc1028@163.com (C.P.); 2 Council of Scientific and Industrial Research (CSIR)-Central Building Research Institute (CBRI), Roorkee 247667, India; debi.kanungo@gmail.com

**Keywords:** soil, polymer, viscosity, reinforcement mechanism, moisture retention, vegetation growth

## Abstract

The significant criterion for evaluating the merits of a new type of high molecular polymer lies in its engineering properties and eco-friendliness. The focus of this study was to determine the effects of preparation conditions on the viscosity of the polyvinyl acetate (PVAc) emulsion, including reaction temperature (*T_r_*), initiator concentration (*C_APS_*), monomer concentration (*C_VA_*), pH value, and degree of dilution (*D_di_*). Based on the results of a series of laboratory tests, the range of viscosity value of PVAc was obtained under different conditions, and one set of viscosity values out of these was applied to soil reinforcement tests. Meanwhile, based on the test results, the engineering properties of PVAc solution were evaluated using strength and moisture retention tests, and the reinforcement mechanism was analyzed using scanning electron microscopy (SEM). In addition, it was proven through a vegetation growth test that the PVAc was eco-friendly.

## 1. Introduction

Adding reinforcement material to soil is internationally considered as an effective method to improve soil properties (i.e., strength performance, permeability behavior, and erosion resistance) [1,2,3,4,5,6]. In general, the reinforcement material is divided into two types, namely, physical reinforcement material and chemical additives. The addition of physical reinforcement material such as wire mesh, geotextile, and other membrane structures can effectively make an improvement in soil engineering performance and is widely used in slope surface protection engineering, as well as highway and railway construction projects [7,8,9,10,11]. It is generally recognized that these methods reinforce soil by utilizing enforcing structures and, on the other hand, they do not modify the physical and chemical properties of the soil [12].

Chemical additives, including traditional and non-traditional additives in reinforced soil, are utilized for modifying soil properties and have been extensively used in soil stabilization [13,14]. The addition of traditional reinforcement materials (inorganic types including cement, fly ash, and their mixture) greatly enhances the properties of reinforced soil (i.e., strength and stiffness) [15,16,17,18]. As is well known, cement/fly ash-treated soil is prone to cracking due to its greater brittleness, thereby resulting in the instability of structures [19,20]. Additionally, the inorganic material inhibits plant growth and has negative effects on the local environment [21,22]. Thus, a better effect will be achieved if a polymer is used. Non-traditional additives such as liquid polymer, resins, acids, silicates, and ions used as a cost-effective and flexible option in soil stabilization have drawn greater public attention in the world [23,24,25,26,27]. Liu et al. [23] discussed the strength behaviors of reinforced sand with an organic polymer using an experimental test method. Mohammed and Vipulanandan [24] investigated the compressive and tensile performances of acrylamide polymer emulsion-treated sulfate contaminated a low plastic soil. Tingle et al. [25] studied the reinforcement mechanism associated with non-traditional additives using laboratory experiments and historical literature. Latifi et al. [26] researched the reinforcement effect of montmorillonite and kaolinite types of clays using a calcium-based non-traditional additive from the macro- and micro-levels. Mousavi et al. [27] displayed the effects of different percentages of polymer used as soil stabilizer to improve the swelling property of expansive soils. The above research achievements demonstrate that non-traditional additives greatly improve the engineering properties of the soil to achieve a specific level of performance. Several factors that influence the performance of polymer-treated soil have been analyzed in detail, including polymer contents, soil types, soil densities, moisture content, and mix design [28,29,30]. The reinforcement mechanism has also been studied [22,31,32], and the mechanism of polymer-reinforced soil is explained as the interaction of the long-chain macromolecules (i.e., the main components of the polymer emulsion) with soil particles’ surfaces. By this physicochemical interaction, the long-chain macromolecules enwrap, connect, and interlink soil particles to form a stable whole and therefore improve the properties of the reinforced soil.

Viscosity is an important parameter for the liquid polymer stabilizer and it plays an important role in the process of soil reinforcement. Meanwhile, the polymer stabilizer can be more convenient in actual operation, and is more economical to transport for a reasonable viscosity value. Furthermore, the eco-friendliness of polyvinyl acetate (PVAc), as a new type of polymer stabilizer, is also an important indicator to distinguish it from traditional reinforcement techniques. Therefore, it is quite essential to study the viscosity and the biological and engineering properties of the PVAc-type of soil stabilizer.

In this study, the effect of reaction conditions on the viscosity of the PVAc emulsion was analyzed in detail, including reaction temperature (*T_r_*), initiator concentration (*C_APS_*), monomer concentration (*C_VA_*), pH value, and degree of dilution (*D_di_*). Based on these test results, a vegetation growth test was conducted. Meanwhile, the engineering properties of the modified soil with different proportions of *C_VA_* were evaluated using unconfined compressive, direct shear, and moisture retention tests, and the reinforcement mechanism was studied using scanning electron microscopy (SEM). The results and associated discussions provide the method and reference for researchers and practicing engineers for the further study of such a polymer.

## 2. Materials and Methods

### 2.1. Polyvinyl Acetate

Polyvinyl acetate (PVAc), an ecological soil stabilizer and an ivory-white liquid, composed of polyvinyl acetate with many long-chain macromolecules and polarity carboxyl groups (–OOCCH_3_), has been used extensively in soil stabilization. PVAc was synthesized by the polymerization reaction of polyvinyl alcohol and vinyl acetate in the presence of persulfate as a free radical initiator in the reaction kettle commonly used for polymerization. PVAc has the following primary advantages: (i) it is easy to produce; (ii) it has a good membrane-forming property, it is non-toxic as well as having no environmental pollution characteristics; and (iii) it can easily achieve the specified level of performance with modification.

In the current study, vinyl acetate (VA), a colorless liquid (supplied by Jinan Yong Chen Chemical Co., Ltd., Jinan, China), was used as a polymerized monomer. This raw material has the advantages of ready availability, being low-cost and non-toxic as well as having no environmental pollution characteristics. 

Ammonium persulfate (APS), a white powder (supplied by Suzhou Bo Chang Chemical Co., Ltd., Suzhou, China), was selected as a free radical initiator in polymerization. 

**Figure 1:**
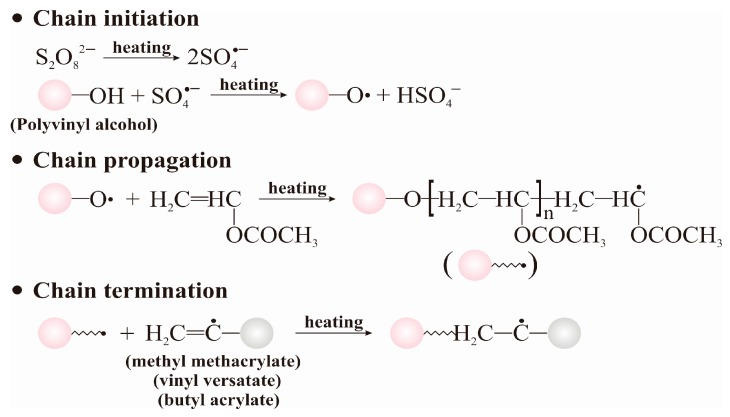
The diagram showing the working principle of vinyl acetate (VA) and polyvinyl alcohol (PVA).

The preparation process of the PVAc emulsion is described in the following steps, and the general procedure adopted for the synthesis of the PVAc emulsion is presented in Figure 1. In addition, the ratio of all these materials in the preparation process is 10:120:1:10:20:10 (polyvinyl alcohol: vinyl acetate: ammonium persulfate: methyl methacrylate: vinyl versatate: butyl acrylate), and the mass ratio of each material is given.

- Step 1: According to the recipe provided above, polyvinyl alcohol was dissolved completely after approximately 3.5 h in distilled water under heating, the temperature being 90 °C. Then, the temperature of the mixture was reduced to 70 °C. Subsequently, ammonium persulfate and vinyl acetate were added to the previous emulsion and continuously stirred for about 20 min.
- Step 2: The methyl methacrylate, vinyl versatate, and butyl acrylate (all supplied by Suzhou Bo Chang Chemical Co., Ltd., Suzhou, China) were mixed evenly and then dripped into the reaction system within 2–3 h. Subsequently, the reaction system was kept at 60–73 °C for 30–60 min.
- Step 3: After the reaction system was cooled down to 50–55 °C, the ammonia, plasticizer, dispersant, and buffer were added to the reaction system and stirred evenly. Then, the reaction system was cooled down to room temperature and the final PVAc emulsion was obtained.

### 2.2. Viscosity Tests

As a new type of polymer reinforcement material, the viscosity property of PVAc is an extremely significant indicator to evaluate its merits. Furthermore, the magnitude of the viscosity is affected by many factors. Therefore, in order to obtain the PVAc with optimal viscosity, five tests with the *T_r_*, the *C_APS_*, the *C_VA_*, the pH value, and the *D_di_* as the variables were performed, respectively, to further determine the best preparation conditions.

#### 2.2.1. Temperature Test

The reaction kettle was used to prepare 8 parts of 1000 mL of the PVAc emulsion according to the above steps, and also the *C_VA_* and *C_APS_* were adjusted to 10% and 0.15%, respectively. In addition, the pivotal point was set with a different *T_r_* in step 1 (*T_r_* = 40 °C, 55 °C, 65 °C, 70 °C, 75 °C, 80 °C, 85 °C, 90 °C) for each preparation. Finally, the viscosity of the emulsion was measured after 12 h.

#### 2.2.2. Initiator Test

The reaction kettle was used as a container to prepare 9 parts of 1000 mL of the emulsion. During preparation of the PVAc emulsion according to the above steps, the *C_VA_* remained the same (10%) but the *C_APS_* was adjusted (*C_APS_* = 0.05%, 0.1%, 0.15%, 0.2%, 0.5%, 1%, 1.5%, 2%, 3%) in each preparation. Furthermore, the *T_r_* in step 1 was controlled according to the optimal *T_r_* as obtained in the temperature test. Finally, the viscosity of the emulsion was measured after 12 h.

#### 2.2.3. Monomer Test

The *C_VA_* was used as the variable and the reaction kettle was again used as the container to prepare the PVAc emulsion. Following the above steps and setting the optimal *T_r_* and *C_APS_* according to the results obtained from the previous two respective tests, the *C_VA_* was adjusted (*C_VA_* = 2.5%, 5%, 7.5%, 10%, 12.5%, 15%) in each preparation. Then, the viscosity of the emulsion was measured after 12 h.

#### 2.2.4. pH Value Test

Generally, the range of the pH value favoring plant growth is 6–7.5. However, the initial PVAc emulsion had a pH value of approximately 2. Therefore, the emulsion was neutralized with NH_3_·H_2_O. The PVAc emulsion was prepared as per the above steps under the optimum conditions obtained in the previous three tests. An appropriate amount of the emulsion was transferred to a beaker after proper dilution and allowed to stand for 12 h. Subsequently, a 5–10 mL portion of NH_3_·H_2_O was added to the beaker and the pH value and viscosity were measured under continuous stirring conditions. The above operation was repeated until the pH value reached approximately 12.

#### 2.2.5. Dilution Test

In order to obtain a PVAc solution suitable for spraying in practical applications, the following operations were performed. First, the pH value of the prepared PVAc emulsion was adjusted to approximately 7, and the emulsion was divided into 24 portions with about 41 g per serving. Then, the emulsion was diluted with water, and the *D_di_* was defined by Equation (1). The *D_di_* values for the 24 proportions of the emulsion varied as follows: 0%, 5%, 10%, 15%, 20%, 25%, 30%, 35%, 40%, 45%, 50%, 60%, 70%, 80%, 90%, 100%, 120%, 140%, 160%, 180%, 220%, 240%, 260%, and 420%. Finally, the viscosity of the solution was measured after thorough mixing:

*D_di_* = (*m’_w_*/*m_w_*) × 100%, (1)

where *D_di_* (%) indicates the degree of dilution, *m’_w_* (g) indicates the mass of additional water added, and *m_w_* (g) indicates the mass of water added during the emulsion preparation.

### 2.3. Soil Reinforcement Tests

To further explore whether the effectiveness of PVAc in enhancing the internal structure of the soil and its eco-friendly properties, the vegetation growth test, strength test, and moisture retention test were conducted in the laboratory. The experimental soil, which was used in the soil reinforcement tests, was taken from the surface of the expansive soil slope along the Ninghuai highway in Jiangsu Province, China. The basic properties of the soil are listed in Table 1.

#### 2.3.1. Vegetation Growth Test

Many existing soil stabilizers have an adverse effect on plant growth. To understand the effects of the PVAc-type soil polymer stabilizer on plant growth, a seed germination test was carried out by adding seeds to the soil. Bermuda grass seeds were selected for this test as that grass is a widely used greening plant in Nanjing city. In order to satisfy the current situation, the concentration of polyvinyl acetate (*C_PVAc_*) was set to 10%, 20%, and 30%, respectively, and water was additionally set as the reference group. First, four culture boxes (length of 20.2 cm, width of 13.6 cm, height of 7.5 cm) were filled with soil. Then, 6 g of Bermuda grass seeds were evenly sprinkled on the surface of the soil in each culture box, and the prepared PVAc solution and water were uniformly sprayed at 3 L/m^2^. After the spray, the culture boxes were placed in indoor conditions with sunshine for cultivation and watered regularly. During the cultivation process, the germination and growth of the seeds were observed and recorded.

#### 2.3.2. Strength Test

In the strength tests, the unconfined compressive strength (UCS), cohesion (*c*), and internal friction angle (*Φ*) of the PVAc-treated soil were measured to evaluate the reinforcement effect. The PVAc solutions with concentrations of 0% (reference group), 5%, 10%, 20%, and 30% were used to understand their effect on the strength of the modified soil. Adopting the static compaction method, unconfined compression specimens (height of 8 cm, diameter of 3.91 cm) and direct shear specimens (height of 2 cm, diameter of 6.18 cm), according to the American Society of Testing Materials Standards D2166-00 and D3080-98, were prepared and according to the corresponding calculations 29 g and 18 g of PVAc solutions were added, respectively. Following the concentration gradient (0%, 5%, 10%, 20%, 30%) in the samples, both types of samples were labeled as S1, S2, S3, S4, and S5, respectively. The specimens thus prepared were placed for curing at 25 °C for 48 h. Then, the unconfined compressive strength and shear strength tests were conducted. During the test of unconfined compressive strength, the rate of the pressure gauge lifting plate of the unconfined pressure gauge was controlled at 2.4 mm/min. During the test of direct shear strength, the loads of the direct shear were set to 50, 100, 200, and 300 kPa, respectively, and the strain rate was kept at 0.8 mm/min. In addition, this experiment was designed with a sample moisture content (*M_c_*) of 17.8% and a dry density (*D_d_*) of 1.7 g/cm^3^. 

#### 2.3.3. Moisture Retention Test

In the moisture retention test, four evaporating boxes were selected and filled up with soil. The surface of the soil in the evaporating boxes was sprayed with 300 mL of PVAc solution with concentrations of 0%, 10%, 20%, and 30%, respectively, and then weighed. Finally, the evaporation boxes were placed in a curing box with a temperature of approximately 40 °C and weighed at regular intervals until the difference in weight between two successive measurements was no greater than 1 g. The evaporativity was given by the following equation:

(2) $$E_{e}=\frac{M_{1}-M_{2}}{M_{0}}\times 100\%,$$

where *E_e_* (%) is defined as the evaporativity of the soil; *M*_1_ (g) is the weight of the soil at the beginning; *M*_2_ (g) is the weight of the soil after a period of time of evaporation; and *M*_0_ (g) is the weight of the dried soil.

## 3. Results

### 3.1. Results of Viscosity Tests

**Figure 2:**
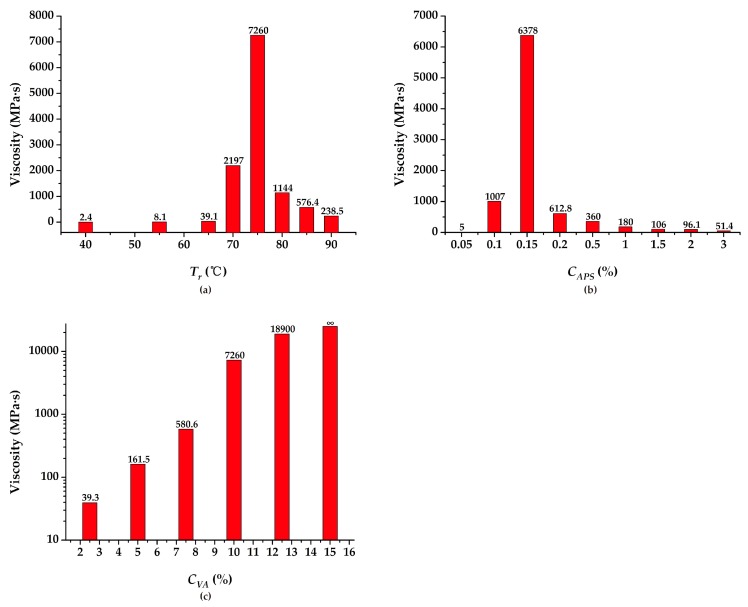
The influence factors of viscosity value: (**a**) reaction temperature (*T_r_*); (**b**) initiator concentration (*C_APS_*); (**c**) monomer concentration (*C_VA_*).

In the viscosity test, the influence factors including *T_r_*, *C_APS_*, and *C_VA_* were studied. The results are displayed in Figure 2. As can be seen, with the increase in *T_r_* and *C_APS_*, the viscosity of the PVAc emulsion increases first and then decreases, as shown in Figure 2a,b. Hence, the optimal *T_r_* and *C_APS_* were selected as 75 °C and 0.15%, respectively. However, as shown in Figure 2c, with the gradual increase of *C_VA_*, the viscosity of the PVAc emulsion increases, endlessly, and finally exceeds the measurement range of the viscometer. In order to confirm the realistic scenario, the optimal *C_VA_* was chosen to be 10%. Based on the above results, the final viscosity value of the PVAc emulsion reached 7260 MPa·s correspondingly. 

**Figure 3:**
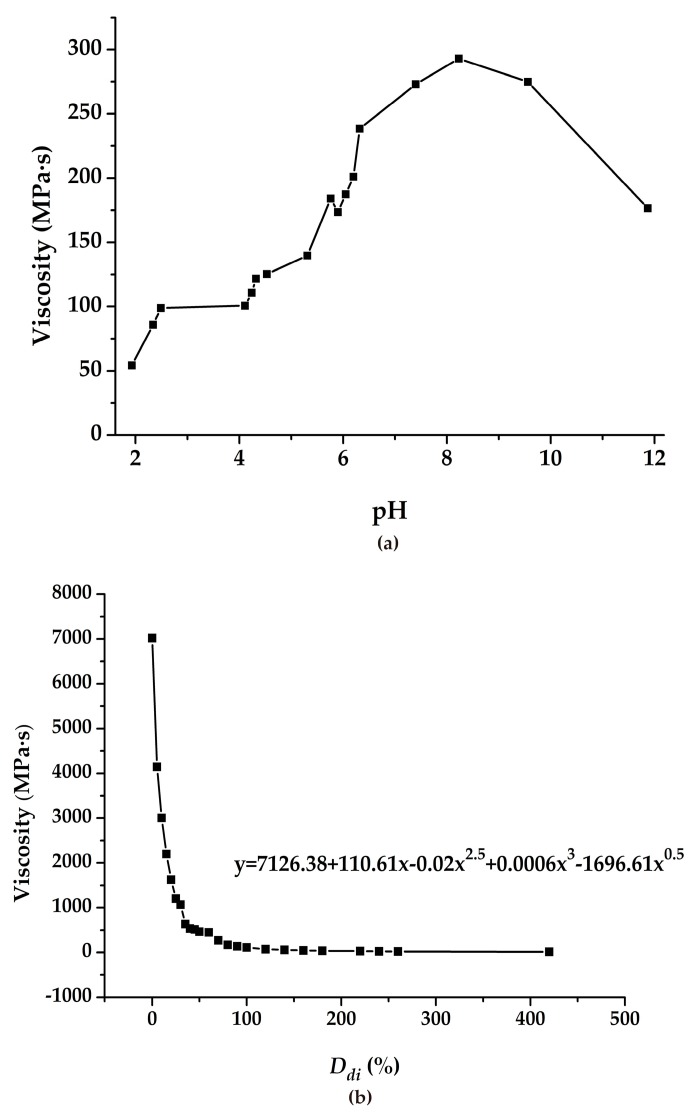
The effect of pH and dilution on the viscosity of polyvinyl acetate (PVAc): (**a**) pH; (**b**) degree of dilution (*D_di_*).

Considering the aspects of actual operation and impact on the environment, the pH test and dilution test were conducted, and the results are shown in Figure 3. As seen in Figure 3a, with the increase in pH of the solution, the viscosity value shows a sharp rise. Then, the viscosity value continues to rise rapidly after a short period of stability. When the pH value is approximately 8.3, the viscosity of the solution reaches a maximum, and afterward, it presents a downward trend. Meanwhile, as can be seen in Figure 3b, with continuous dilution of the emulsion, its viscosity value first drops dramatically and then tends gradually to zero. In particular, at 50% *D_di_*, the emulsion viscosity attains merely a fourteenth of the initial viscosity. Moreover, according to the emulsion viscosity dilution curve in Figure 3b, the change of viscosity confirms that y = 7126.38 + 110.61x − 0.02x^2.5^ + 0.0006x^3^ − 1696.61x^0.5^.

According to the results of the viscosity test, the range of the viscosity value change of PVAc can be seen in Table 2. Meanwhile, considering the aspect of practical application, the optimal viscosity value was selected and applied to the subsequent tests. Various parameters of PVAc are shown in Table 3.

### 3.2. Results of Soil Reinforcement Tests

#### 3.2.1. Vegetation Growth Test

**Figure 4:**
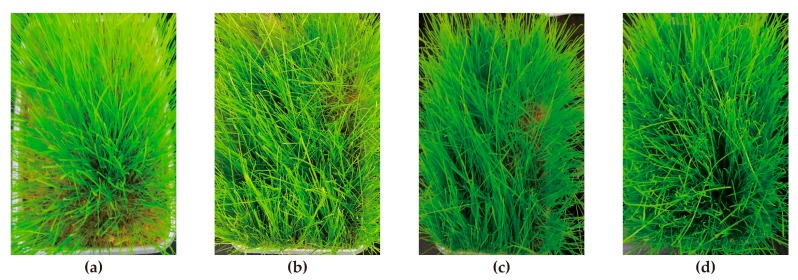
The images after germination: (**a**) the concentration of polyvinyl acetate (*C_PVAc_*) is 0%; (**b**) the *C_PVAc_* is 10%; (**c**) the *C_PVAc_* is 20%; (**d**) the *C_PVAc_* is 30%.

In the vegetation growth test, the time and situations of germination and the situations of soil structure were studied. As can be seen from Table 4, the PVAc-type soil polymer stabilizer does not have any adverse effect on the growth of the plants. By spraying the PVAc solution, the germination and growth of Bermuda grass seeds are better than those without it. According to Figure 4, as the addition of *C_PVAc_* to the soil increases, the germination rate becomes higher. In addition, when the *C_PVAc_* was 30%, an earlier germination by one day occurred and had a higher germination rate than the other groups. Meanwhile, with the higher amount of PVAc solution in the test sample, the soil condition was better.

#### 3.2.2. Strength Test

In the strength test, the change of UCS, *c*, and *Φ* of the modified soil were studied, and the results are shown in Table 5. By comparison with the reference group, it can be observed that the UCS of the modified soil has obviously enhanced and the maximum reached 233.3 kPa, which is acout 2.06 times the strength of the reference sample. At that time, the corresponding viscosity was 3003 MPa·s.

Under the curing conditions for 48 h, the cohesion of the reference group and S5 reached 260 kPa and 384.4 kPa, respectively. It clearly shows an increase of approximately 25–32% in cohesion of the modified soil compared to that of the reference group. However, the internal friction angle of the modified soil has no distinct change and remains basically the same as that of the reference group at the internal friction angle of approximately 58.5° (±3°).

**Figure 5:**
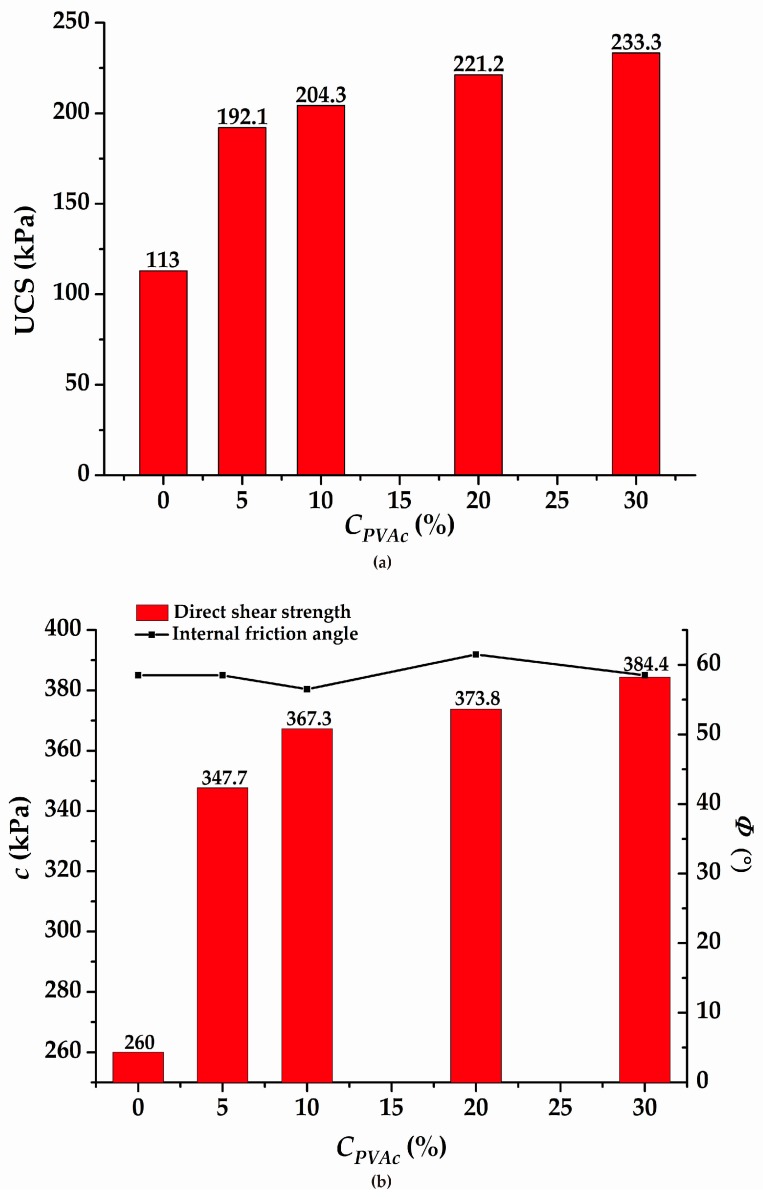
The variation of strength with *C_PVA__c_*: (**a**) unconfined compressive strength (UCS) and (**b**) cohesion parameters.

Figure 5 illustrates more clearly the change trend of UCS, *c*, and *Φ* with the modified *C_PVAc_* in the modified soil specimens. A great influence on the UCS and *c* is observed with or without the addition of the PVAc to the specimens. Meanwhile, as the *C_PVAc_* increases, the ability of the specimens to resist both the compression and shear obviously increases. Furthermore, there is no significant change in the *Φ* value with the increase in *C_PVAc_*.

#### 3.2.3. Moisture Retention Test

**Figure 6:**
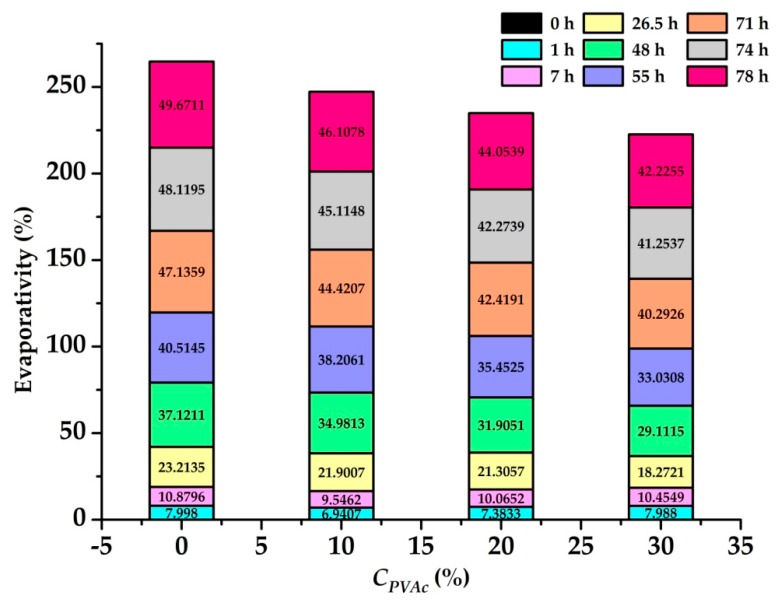
The relationship between C*_PVAc_* and evaporativity.

In the moisture retention test, the relationship between evaporativity and time of samples with different solution concentrations was studied, and the results are shown in Figure 6. As the *C_PVAc_* increases gradually, the evaporativity continuously reduces. This indicates that the higher the *C_PVAc_*, the stronger the water retention property of the soil. In particular, the evaporativity values of the four evaporating boxes are roughly the same within approximately 10 h. Then, after about 20 h, the difference in the moisture retention property gradually emerges. Moreover, the spraying of a 30% polymer solution reduced the soil evaporativity by approximately 7.45% compared with the spraying of water, and the minimum value of evaporativity was reached at approximately 42.2%.

## 4. Discussion

### 4.1. Vegetation Growth Characteristic

According to the analysis, the reason behind the growth of the plants is mainly the improvement in the water stability of the modified soil particles. Thus, the structure of the soil and its physical properties such as porosity, gas permeability, and water permeability are improved. Meanwhile, the loss of moisture and nutrients is reduced and the heat preservation and moisturizing effect are produced. Consequently, it further promoted the growth of the plants. Thus, it can be seen that the PVAc-type soil polymer stabilizer not only has no adverse impact on the growth of the plants, but also plays a promoting role to some degree.

### 4.2. Strength Characteristic

**Figure 7:**
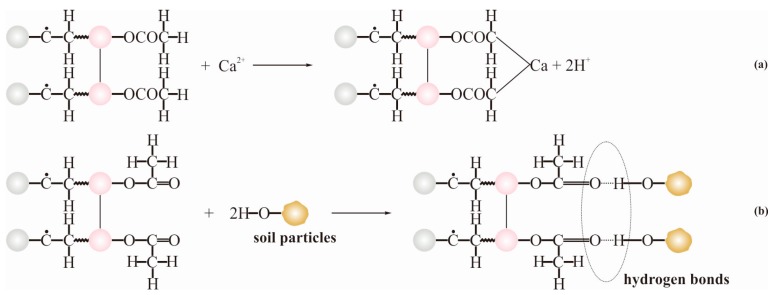
The reaction mechanism of the soil particles and the PVAc emulsion: (**a**) the displacement reaction of alkaline metal ions; (**b**) the reaction of hydrogen bonds formation.

Both UCS and *c* of the modified soil are essential parameters for assessing the effect of the stabilizer. Generally, the mechanism, by which organic polymers act on soils, involves a series of chemical and physical reactions. When a PVAc solution acts on cohesive soil, the polarity carboxyl (–OOCCH_3_) hydrophilic group on the macromolecular chain conducts a displacement reaction with the alkaline metal ions in the soil that can be seen in Figure 7a. Once the cations on the surface of the soil particles are displaced, the thickness of the electric double layer becomes thinner, and the potential decreases immediately. Therefore, the attraction between the soil particles increases and the aggregation is promoted. Furthermore, except for the displacement reaction, the hydrogen bonds can be formed owing to the reaction between the hydroxyl group on the surface of the soil particles and the polarity carboxyl group. This process is shown in Figure 7b. Meanwhile, a growing number of hydrogen bonds make the enhancement in UCS and *c* of the modified soil with the augmentation of the PVAc solution.

**Figure 8:**
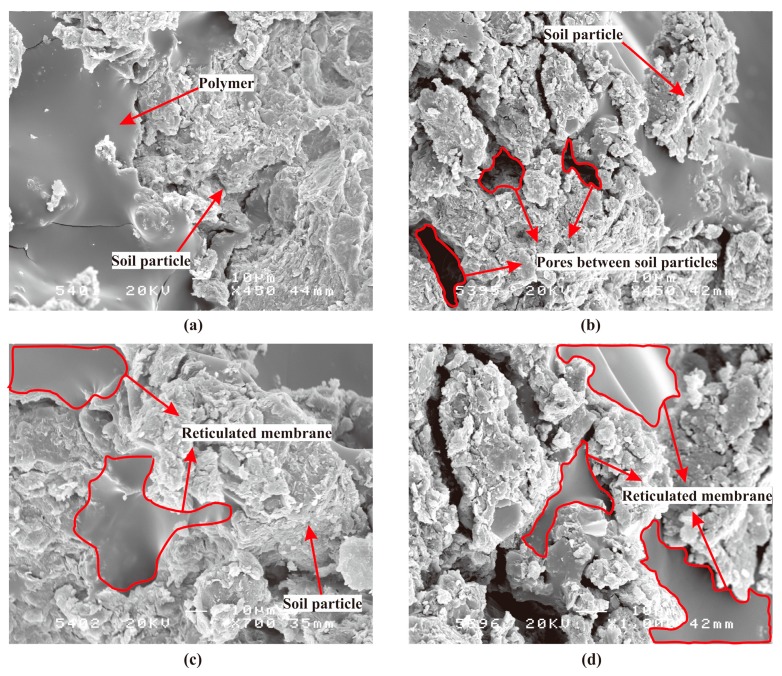
The scanning electron microscopy (SEM) micrographs of reinforced soil: (**a**) 450 times magnification; (**b**) 450 times magnification; (**c**) 700 times magnification; (**d**) 1000 times magnification.

With curing for 48 h at room temperature in addition to the above chemical reactions, a series of physical reactions between the PVAc emulsion and the soil particles occurred to form a certain network membrane structure with elasticity and glutinousness by mutual diffusion, penetration, and entanglement. This process sufficiently filled the existing pores between the soil particles. Meanwhile, the soil particles were glued well and the overall strength was increased significantly because of the effect of the reticulated membranes. Moreover, the reduction in water content by curing the specimens consequently reduced the thickness of the hydration film and the distance between the soil particles. At that point, the contact area and the surface tension of the capillary water between the soil particles had an obvious enhancement. This also contributed to the improvement in the soil strength. The scanning electron microscopy image (Figure 8) further supports such evidence clearly.

### 4.3. Moisture Retention Characteristic

**Figure 9:**
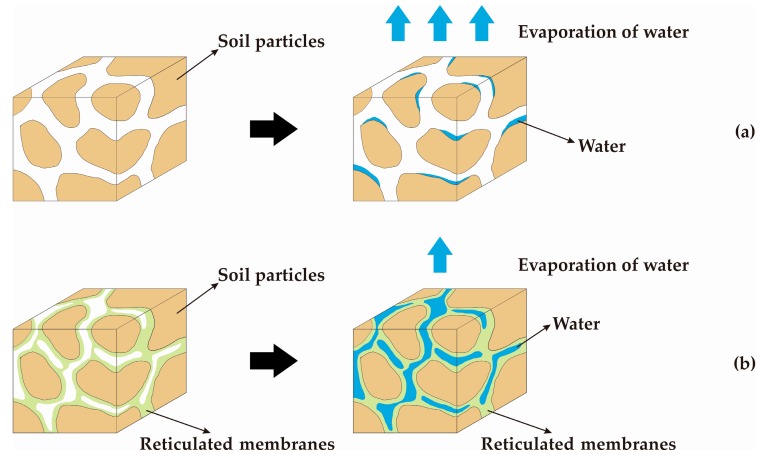
The schematic diagram of the moisture retention property: (**a**) soil; (**b**) soil reinforced by PVAc.

While the PVAc solution reacts with the metal cations on the surface of the soil to produce a host of replacement reactions and forms the mass of hydrogen bonds with the hydroxyl group, it also undergoes a series of physical reactions with the soil particles to form the reticulated membrane structures with elasticity and glutinousness by interpenetrating, diffusing, and cementing. As shown in Figure 9b, it is obvious that the reticulated membranes formed by adding the PVAc solution tightly glue the soil particles together, thereby completely filling the existing pores between the soil particles and increasing the strength of the overall structure. Its water retention capacity dramatically improves on account of this change in the internal structure and further locks up more moisture. Conversely, it can be seen from Figure 9a that the internal structure of the soil without the PVAc solution is still very loose and contains a certain number of pores between the soil particles. As time goes by, the water inside it will gradually dissipate to the surrounding area.

### 4.4. Application of Polyvinyl Acetate

Global climate and environmental changes have caused a series of problems, such as soil erosion, slope instability, and subgrade damage, etc. As a new type of polymer soil stabilizer, PVAc, has been successfully investigated and developed, and it could effectively solve the above problems. It was shown through a series of laboratory tests that PVAc could effectively change the physical structure of the soil and improve the soil mechanical strength and moisture retention, among other things, without affecting vegetation growth. Table 6 shows the application of PVAc in different situations.

The application methods in Table 6 are suggestions, for which appropriate methods can be developed according to the specific situation. In addition, the concentration range in Table 6 is a reference value, which can be adjusted according to the specific situation. The cost range in Table 6 is a reference value calculated based on the application method and concentration. Song et al. [32] studied the application of PVAc to a gentle slope (slope < 35°) reinforcement on a soil slope located along the Ninghuai highway, Jiangsu, China. The application of PVAc in other situations will be studied in the future.

## 5. Conclusions

In this paper, a series of laboratory tests were carried out to determine the effect of reaction temperature, initiator concentration, monomer concentration, pH value, and degree of dilution on the viscosity of a polymer emulsion. The mechanism of these factors affecting the viscosity of the polymer emulsion was also briefly analyzed. Based on these test results, the engineering properties of the PVAc solution were evaluated using strength and moisture retention tests and were studied with scanning electron microscopy. Furthermore, the non-biotoxic property of PVAc was proven using biological tests. The results obtained in this study can be summarized as follows:

1. Through a series of laboratory tests, it was shown that *T_r_*, *C_VA_*, and *C_APS_* obviously affected the viscosity change of the PVAc emulsion under the reaction conditions. With the increase in *T_r_*, *C_VA_*, and *C_APS_*, the emulsion viscosity showed an increasing trend initially and then a decreasing trend, which mainly depended on whether the free radical generated by the initiator reached the optimal amount. When such a condition was reached, the monomer molecule was continuously connected with the free radicals to carry out the chain growth reaction. Meanwhile, the change of pH value and *D_di_* also affected the viscosity value of PVAc. After a comprehensive series of laboratory tests were performed, a range of viscosity values for PVAc was obtained under different conditions. The *T_r_*, *C_APS_*, *C_VA_*, pH value, and *D_di_* were 40–90 °C, 0.05–3%, 2.5–15%, 1.93–11.87, and 0–420%, respectively. Correspondingly, the range of viscosity values were 8.8–7260 MPa·s, 5–6378 MPa·s, 39.23–+∞ MPa·s, 217–1172 MPa·s, and 12.74–7020 MPa·s.
2. It was observed from the strength tests that the compressive strength and cohesion of the modified soil sample underwent an obvious improvement as compared with the reference group. Simultaneously, as the *C_PVAc_* persistently increased, the UCS and *c* both witnessed a correspondingly continuous enhancement. This can be attributed to the effect of the PVAc solution on the soil to form a series of chemical bonds and elastic mucosa between the soil particles. Then, the maximum values of UCS and *c* were observed as 233.3 kPa and 384.4 kPa, respectively. The corresponding viscosity value reached 3003 MPa·s at that time. However, a change in the internal friction angle of the samples was not distinctly observed.
3. It can be inferred from the water retention test that as the concentration of the added PVAc solution increased, the moisture retention property of the soil increased correspondingly. This is mainly due to the formation of a large number of reticulated membrane structures with elasticity and glutinousness when the PVAc solution reacted on the soil. Hence, the pores between the soil particles were sufficiently filled with the moisture. From the test results, it was also shown that the spraying of a 30% PVAc solution reduced the soil evaporativity by approximately 7.45% compared with water.

## Figures and Tables

**Table 1:** The basic properties of the soil.

**Basic Properties**	Liquid limit (%)	Plastic index *I_p_*	Specific gravity	Optimum water content (%)	Maximum dry density *ρ_d_* (g/cm^3^)	Free swelling ratio *δ_ef_* (%)
**Values**	52.6	19.7	2.73	15.4	1.73	53

**Table 2:** The range of viscosity values of PVAc.

Conditions	*T_r_*^1^ (°C)	*C_APS_*^2^ (%)	*C_VA_*^3^ (%)	pH Value	*D_di_*^4^ (%)
	40–90	0.05–3	2.5–15	1.93–11.87	0–420
**Viscosity (MPa·s)**	8.8–7260	5–6378	39.23–+∞	217–1172	12.74–7020

^1^ is defined as the reaction temperature; ^2^ is defined as the concentration of initiator; ^3^ is defined as the concentration of monomer; ^4^ is defined as the degree of dilution.

**Table 3:** The basic parameters of the PVAc solution.

Viscosity (MPa·s)	*C_VA_*^2^ (%)	*C_APS_*^3^ (%)	pH Value	Water Absorption (%)	Gel Fraction (%)
716.1–904.8 ^1^	10	0.15	6–7.5	34	1.48

^1^ The viscosity value is measured when the concentration is diluted to 30% and the temperature is 25 °C; ^2^ is defined as the concentration of monomer; ^3^ is defined as the concentration of initiator.

**Table 4:** The situations of germination and the variation of soil structure.

The Concentration of Polyvinyl Acetate (%)	Germination Time (d)	The Situations of Germination ^1^	The Situations of Soil Structure ^2^
0	4	1	1
10	4	1	2
20	4	2	3
30	3	3	3

^1^ The larger the value, the higher the germination rate. ^2^ The larger the value, the more complete is the soil.

**Table 5:** The results of the strength tests.

Number	*M_c_*^1^ (%)	*D_d_*^2^ (g/cm^3^)	*C_PVAc_*^3^ (%)	Viscosity (MPa·s)	UCS ^4^ (kPa)	*c*^5^ (kPa)	*Φ*^6^ (°)
S1	17.8	1.7	0	0.0	113.0	260.0	58.5
S2	17.8	1.7	5	168.6	192.1	347.7	58.5
S3	17.8	1.7	10	524.5	204.3	367.3	56.5
S4	17.8	1.7	20	2197	221.2	373.8	61.5
S5	17.8	1.7	30	3003.0	233.3	384.4	58.5

^1^ is defined as the moisture content of the samples; ^2^ is defined as the dry density of the samples; ^3^ is defined as the concentration of polyvinyl acetate; ^4^ is defined as the unconfined compressive strength of the modified soil; ^5^ is defined as the cohesion of the modified soil; ^6^ is defined as internal friction angle of the modified soil.

**Table 6:** The application of PVAc in different situations.

Situation	Application Method	Concentration Range	Cost Range (dollars/m^2^)
Gentle slope (slope < 35°)	Spraying ^1^	20–25%	30–37.5
Medium slope (slope: 35–65°)	External-soil spray seeding ^2^	20–25%	40–47.5
Steep slope (slope > 65°)	External-soil spray seeding + Steel net ^3^	25–35%	46–53.5
Subgrade	Mixing + Compaction ^4^	15–20%	30–37.5
River bank	Spraying	20–25%	30–37.5
Mine restoration	External-soil spray seeding + Steel net	25–35%	46–53.5

^1^ PVAc is diluted and sprayed directly on the slope. ^2^ A mixture of PVAc diluent and soil is sprayed on the slope. ^3^ After laying a steel net on the slope surface, a mixture of PVAc diluent and soil is sprayed on the slope. ^4^ After mixing the PVAc diluent with the soil, the mixture is laid on the roadbed and compacted.
